# Mitochondrial complex I modulation targets metabolic plasticity in breast cancer cells

**DOI:** 10.1186/2049-3002-2-S1-P59

**Published:** 2014-05-28

**Authors:** V Krishnan Ramanujan, Qijin Xu, Eva Biener-Ramanujan

**Affiliations:** 1 Surgery, Cedars-Sinai Medical Centre, Los Angeles, CA 90048, USA; 2 Medicine, Cedars-Sinai Medical Center, Los Angeles, CA 90048, USA

## Background

Cancer cell transformation entails significant alterations in intracellular metabolic pathways one of which is the aerobic glycolysis phenotype [1]. The original formalism of this phenotype was hypothesized by Otto Warburg in 1950s and main tenet of his hypothesis was that mitochondrial dysfunction in cancer cells leads to aerobic glycolysis phenotype [2]. It is not clear in the field if mitochondrial dysfunction is a necessary condition for observing the aerobic glycolysis phenotype and further it is also not known if this “metabolic switch” phenotype is reversible. We recently showed that mitochondrial dysfunction (generated by gene silencing of catalytic subunit of mitochondrial complex I in transformed cells indeed can induce metabolic switch phenotype [3]. We further demonstrated that this can be reversed for moderate mitochondrial dysfunction models. In the present study, we ask if we can achieve tumor control in preclinical animal models by systematic modulation of mitochondrial complex I function via metabolic adaption to clinically relevant pharmacological modulators of mitochondria in breast cancer cells.

## Materials and methods

We used two human breast cancer cell lines (MDA-MB-231 & MDA-MB-453) in this study where we cultured these cell lines in the presence of mitochondrial complex I inhibitor (rotenone) inhibitor for 25 generations thereby creating metabolically adapted cell lines counterparts. Metabolic analysis (*in vitro* and *in vivo*) of the parental and modified cancer cells was carried out.

## Results

Analysis of metabolic status in the isogenic parental and metabolically modified adaptive breast cancer cells show an overall improvement in mitochondrial function and a reduction in metabolic switch phenotype. *In vivo* analysis of tumor xenografts revealed that metabolically modified MDA231 cells displayed a ~50% reduction in tumor growth/volume accompanied by a reduced *in vivo* proliferation rate in comparison with the parental MDA231 cells thereby confirming the physiological relevance of metabolic adaptation in preclinical animal models. In conclusion, long-term metabolic adaptation to mitochondrial complex I modulators can be a unique and novel strategy for achieving tumor control *in vitro* and *in vivo*.
